# Complementing clinical cancer registry data with patient reported outcomes: A feasibility study on routine electronic patient‐reported outcome assessment for the Austrian Myelome Registry

**DOI:** 10.1111/ecc.13154

**Published:** 2019-08-29

**Authors:** Monika Sztankay, Lucia Neppl, Lisa M. Wintner, Fanny L. Loth, Wolfgang Willenbacher, Roman Weger, Walpurga Weyrer, Michael Steurer, Gerhard Rumpold, Bernhard Holzner

**Affiliations:** ^1^ Medical University of Innsbruck Innsbruck Tirol Austria; ^2^ Psychiatry II Innsbruck University Hospital Innsbruck Tirol Austria; ^3^ University of Innsbruck Innsbruck Tirol Austria; ^4^ Internal Medicine V: Haematology & Oncology Innsbruck University Hospital Innsbruck Tirol Austria; ^5^ Oncotyrol–Center for Personalized Cancer Medicine Innsbruck Tirol Austria

**Keywords:** cancer registry, clinical routine, electronic patient‐reported outcome monitoring, health‐related quality of life, implementation, multiple myeloma

## Abstract

**Objectives:**

Routinely assessed patient‐reported outcomes (PROs), such as quality of life (QOL), are important to supplement clinical cancer data but requires rigorous implementation. This study aims at depicting the implementation procedure and evaluating the feasibility of routine electronic PRO monitoring (ePRO) for collecting data supplementing the Austrian Myeloma Registry (AMR).

**Methods:**

Integration of ePRO monitoring into clinical routine was planned according to the Replicating Effective Programs framework. QOL data were assessed regularly during treatment and aftercare at the hematooncological outpatient unit at the Medical University of Innsbruck with the EORTC QLQ‐C30/ +MY20 and the EQ‐5D‐5L. Feasibility and usability testing were performed via a multimethod approach.

**Results:**

Within the first year, 94.4% of the MM patients (*N* = 142, mean age 65.4, *SD* 11.8, 60% male) provided 748 PRO assessment time points overall. Patients and clinicians were satisfied with ePRO monitoring and indicated no to little disruption in clinical routine. Patient preference on assessment time points and completion frequency became evident.

**Conclusions:**

Complementing the AMR with ePRO data proved to be feasible. Our findings provide useful insights for healthcare providers considering introducing ePRO monitoring to their units for informing clinical registries as well as individualised feedback to patients alike.

## 1 INTRODUCTION

Due to increased availability of once so‐called novel agents in first‐line treatment (bortezomib and lenalidomide), the prognosis for patients suffering from multiple myeloma (MM) has been considerably improved within the last decade (Kumar et al., 2014; Mey et al., 2016). For patients with advanced disease, likewise, the number of available treatment options, similar in safety and clinical effectiveness, has substantially increased (Anderson, 2016; Orlowski & Lonial, 2016). With regard to the growing complexity of treatment decisions and prolonged survivorship in a still incurable disease following a highly protracted course characterised by a high symptom burden, the value of patient‐reported outcomes (PROs), such as quality of life (QOL), supplementing clinical data for the comprehensive haemato‐oncologic care and research is increasingly recognised (Efficace, Gaidano, & Lo‐Coco, 2017). With the introduction of all oral regimes (e.g., Ixazomib based) and immunotherapy into MM treatment regimes, questions like the respective subjective preference towards alternative treatment approaches and adherence attitudes are of paramount importance.

PROs are defined as ‘any report of a patient's health condition coming directly from the patient, without interpretation by a clinician or anyone else’ (FDA, 2006). To realise their potential in informing oncological treatment and research, PROs need to be linked to patients’ clinical data such as those collected systematically by cancer registries. PROs can contribute information across the spectrum of cancer registry purposes by augmenting the evaluation of adverse events, determining comparative effectiveness of oncological treatment strategies or predicting meaningful clinical outcomes such as survival. PRO data collected prospectively can be used in clinical routine to support decision‐making and patient‐centred care (Dubois et al., 2006; Efficace et al., 2012; Quinten et al., 2011; Wood et al., 2016). For regulators, PRO data provide crucial information for quality assurance, the allocation of health resources and the development as well as targeted provision of support services (Parkin, 2006).

The traditional role of cancer registries has already expanded beyond the monitoring of disease parameters such as cancer prevalence, mortality and survival to monitoring factors that influence clinical outcomes as well as the treatment process (Parkin, 2006). A number of studies have called for extension of the use of cancer registries to include collection of PROs (Ashley et al., 2013; Santanello, Largent, Myers, & Smalley, 2018; van de Poll‐Franse et al., 2011). Up to now, few promising initiatives supplementing cancer registries with PRO data have been reported (Ashley et al., 2013; Kent et al., 2016). While the registration data reporting is an established practice, there are no standards for how to implement PRO assessment procedures in routine cancer registration. Findings, however, offer strong support for the premise that systematic and rigorous implementation is associated with better outcomes regarding the success and sustainability of any intervention (Durlak & DuPre, 2008; Efficace et al., 2017).

Therefore, the aim of this study was to depict the implementation procedure and to evaluate the feasibility and usability of routine ePRO monitoring for integrating patient‐reported QOL data with data of the Austrian Myeloma Registry (AMR) at a single participating centre. Additionally, differences in attitudes towards routine ePRO assessment between new and more experienced users are evaluated.

## 2 METHODS

### 2.1 The Austrian myeloma registry

The AMR was established in 2008 (comprising retrospective inclusion of patients diagnosed back to the year 2000) under the auspices of the Austrian Society for Hematology & Oncology (OeGHO; http://www.oegho.at), operated by Oncotyrol (http://www.oncotyrol.at/amr/). The software is maintained by Evaluation System Development (https://ches.pro). The registry is an online database, to be used in conjunction with respective clinical information systems, for documenting clinical characteristics and outcomes of MM treatment in Austria (and now also internationally) providing longitudinal ‘real‐life’ data from a large patient population across the whole treatment trajectory (http://www.myeloma-registry.com). All patient records and information are anonymised and de‐identified prior to analysis, compatible with the European data protection regulations. In Austria, 17 centres are included into the AMR, with an overall of 1.192 patients. Being one of the largest participating centres contributing data to the AMR, the University Hospital of Innsbruck has included approximately 353 MM patients up to now. Inclusion of register‐naive patients is continuously ongoing. Ethical approval was obtained from the Ethical Committee of the Medical University of Innsbruck (study number AN3252 266/4.2 386/5.14).

### 2.2 Implementation procedure for integrating regular ePRO monitoring

**Figure 1:**
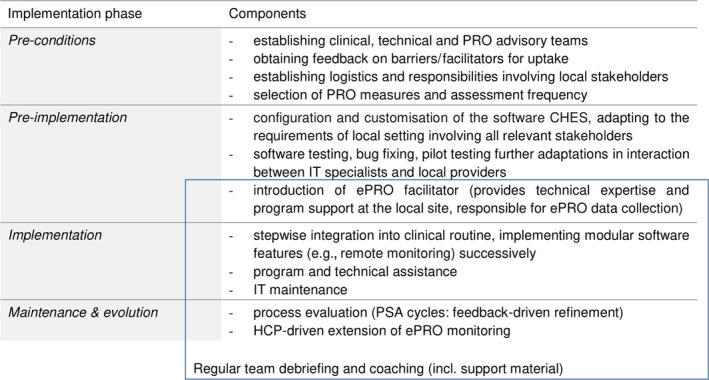
Replicating Effective Programs phases of implementation of ePRO monitoring into clinical workflow

Implementation of routine ePRO monitoring used a pragmatic strategy on the basis of the Replicating Effective Programs (REP) framework (Kilbourne, Neumann, Pincus, Bauer, & Stall, 2007) leveraging local healthcare providers’ initiative and outside ePRO expertise (Wintner et al., 2016). REP focuses on standardisation of the implementation of healthcare interventions into routine care settings and consists of four phases. Its participatory approach involves all relevant stakeholders (detailed in Figure 1).

The first phase, *pre‐conditions*, encompassed the initiative of the AMR executive on supplementing the registry with PRO data. Decisions on the choice of EORTC PRO measures (as suggested e.g., in Huebner et al., 2014) and software were made in consultation with the PRO advisory group in the initial phase. A common understanding, taking into account the requirements of the local leadership as well as retentions of the on‐site staff, was established concerning the rationale of ePRO monitoring and possible clinical applicability of the data. Logistics with the least possible disruption of the existing clinical workflow were planned and set‐up in discussion with the relevant stakeholders.

In the second phase, *pre‐implementation,* the software Computer‐based Health Evaluation System (CHES, © ESD 2018) was customised to fit local requirements (Holzner et al., 2012; e.g., interface to the clinical information system, graphical user interface, adding reference values calculated based on local reference cohorts and thresholds for clinical relevance (Giesinger et al., 2016, Wintner et al., 2016). To minimise logistic burden for the local healthcare professionals (HCPs), a designated staff member (herein, the *ePRO facilitator*) was introduced to the unit to support PRO data collection with designated time and technical expertise.

During the third phase, *implementation*, ePRO monitoring was initiated at the outpatient unit. Regular multidisciplinary team meetings were held along the whole implementation process to obtain suggestions for improvement and feedback on technical and logistic issues as well as clinical application of ePRO data of individual patients. The staff received supportive material on the use of CHES and theoretical background on ePRO monitoring.

In the fourth phase, *maintenance and evolution*, patient and staff surveys were conducted and during team meetings further uses of ePRO data and possible extensions of the monitoring were discussed. Given the scope of this paper, the results of the process evaluation in the final phase will be described in more detail herein.

### 2.3 Sample

Patients were recruited at the haemato‐oncological outpatient unit at the Department of Internal Medicine V (Haematology and Oncology) at the University Hospital of Innsbruck. The outpatient unit is staffed with five haemato‐oncologists, one to two changing residents, eleven nurses and two further staff members. Patients were eligible for inclusion to the AMR as well as the ePRO assessment if they were (a) over 18 years of age, (b) diagnosed with multiple myeloma, (c) had no overt cognitive impairment, (d) were German literate and (e) provided written informed consent.

### 2.4 Assessment instruments

#### 2.4.1 PRO measures

The modular EORTC Quality of Life system was chosen to assess QOL in MM patients. The *EORTC QLQ‐C30* (Aaronson et al., 1993) is an internationally validated and widely used cancer‐specific QOL measure, comprising five functional scales (physical, role, emotional, social and cognitive), three symptom scales (fatigue, nausea & vomiting and pain), six single items (dyspnoea, insomnia, appetite loss, constipation, diarrhoea and financial difficulties) and a scale for the patient's global health status/QOL. The core questionnaire was supplemented by the *EORTC QLQ‐MY20* (Stead et al., 1999) addressing issues relevant to myeloma patients. It consists of two symptom scales (disease symptoms, side effects of treatment), one functioning scale (future perspective) and one single item to evaluate body image. The EORTC QOL system was supplemented with the *EuroQol‐5D‐5L* (*EQ‐5D‐5L*; Brooks, 1996), which is a standardised generic preference‐based measure for estimating health state utility values in five dimensions of health including mobility, self‐care, usual activities, pain/discomfort, and depression/anxiety.

#### 2.4.2 Feasibility and usability testing

The ‘Fit between Individuals, Task and Technology’ (FITT) framework by Ammenwerth and colleagues (Ammenwerth, Iller, & Mahler, 2006) was adopted to guide the feasibility and usability assessment from the patient and provider perspective (Steele Gray et al., 2016). The FITT framework suggests that adopting a new information technology (IT) system in the clinical environment depends on the fit between the attributes of the users (e.g., comfort with technology, motivation), attributes of the technology (e.g., usability), and attributes of the clinical tasks and processes (e.g., task complexity). Herein, feasibility and usability testing comprise the assessment of the following parameters: resources required to complete tasks, for example delay in clinical practice, administrative burden (*efficiency*), the ability to complete tasks completely and accurately, for example reported errors, required assistance, difficulties while using the tool (*effectiveness*), *learnability* (e.g., increase in user skills after multiple assessments) and *user satisfactio*n.

A multimethod approach was applied including the following measures:

1. *Feasibility survey*. To assess the patients’ and the HCPs’ perspectives, a survey was designed including questions asking for the responders’ perspective on the feasibility and usability of the ePRO monitoring tool, aspects of integration into clinical workflow, administrative burden of assessment, need for assistance, reported learnability and attitudes towards use of PRO data in routine care. Twenty patients were consecutively enrolled to complete the survey after their baseline ePRO assessment (hereafter, ‘ePRO‐naïve group’) and 20 patients after multiple (at least 5) ePRO assessment time points (hereafter, the ‘ePRO‐experienced group’). HCP feedback was collected from all haemato‐oncologists and nurses who wished to participate.
2. *On‐site performance*. Observational notes on CHES performance and assessment procedure, such as technical or logistic issues, were assessed on‐site by the ePRO facilitator.
3. *Additional feasibility outcomes* included patient participation, response rate, dropouts and adherence with questionnaire completion (incl. reasons for non‐completion, percentage of missing items).

### 2.5 Infrastructure and software for ePRO assessment

**Figure 2:**
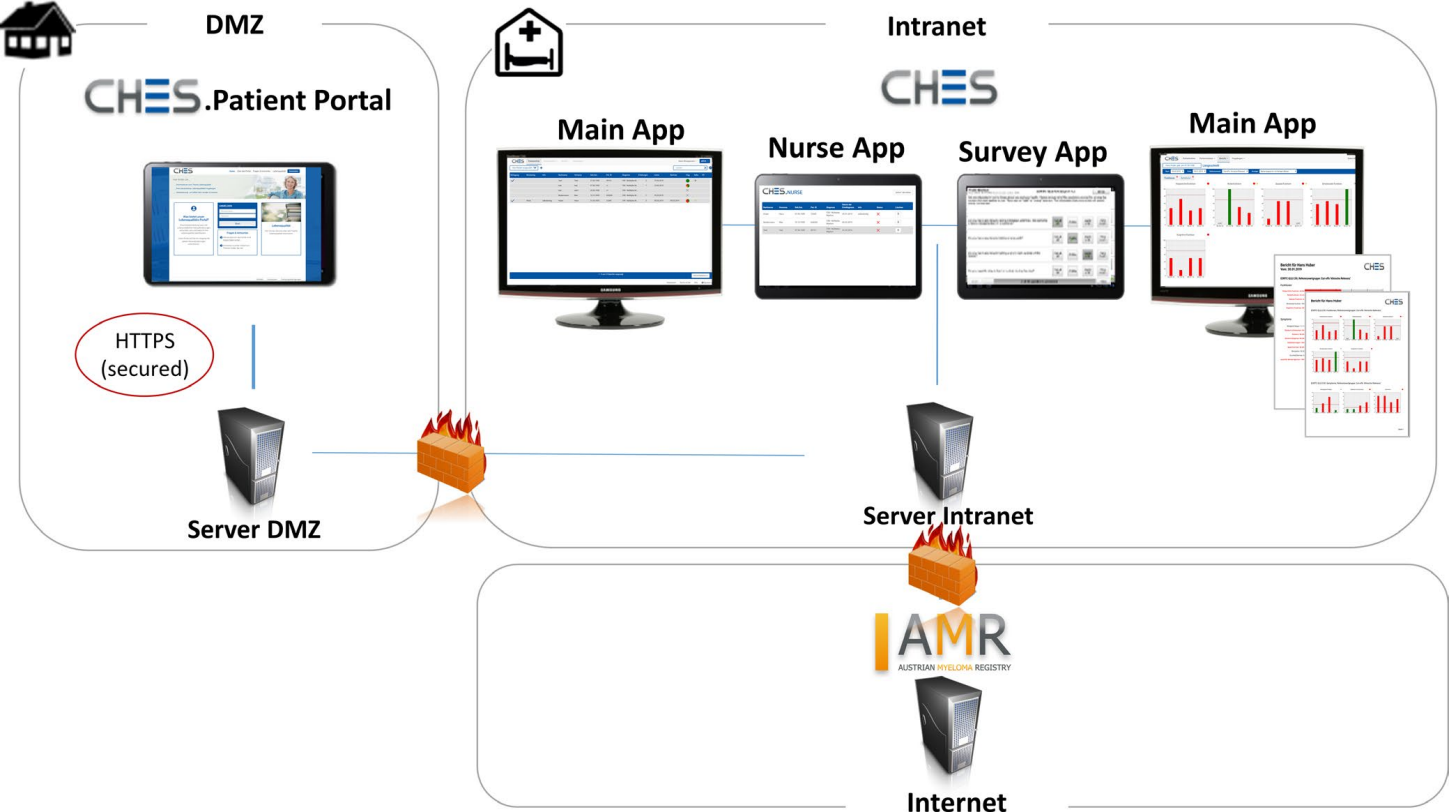
Computer‐based Health Evaluation System (CHES) Infrastructure

The ePRO infrastructure was provided by the software CHES. CHES is a software tool for the electronic administration, collection, calculation and graphical presentation of PROs and medical data (Holzner et al., 2012; © ESD 2017). Healthcare professionals can access the questionnaire results immediately after questionnaire completion on any workstation within the hospital network via the web browser (cf. Figure 2). In addition, CHES provides data exchange interfaces to connect with clinical information systems (e.g., HL7) and cancer registries (e.g., AMR). Data are transferred via a secure interface, linked by an identifier.

### 2.6 Assessment procedure of ePRO monitoring

The ePRO facilitator consecutively recruited patients for ePRO monitoring during their appointments at the outpatient unit. After informed consent for inclusion to the AMR, they were asked for their consent to participate in clinical ePRO monitoring at their clinical appointment. ePROs were assessed at any given post‐diagnostic time point (according to treatment schedule) using tablet PCs running CHES. The ePRO facilitator introduced participants to routine QOL monitoring, the use of the device and provided assistance if necessary. Participants were invited to contribute comments and recommendations concerning the procedure.

### 2.7 Statistical analyses

Sample characteristics are presented as frequencies, percentages, ranges, means and standard deviations. Mann–Whitney *U* test and chi‐squared test were applied for comparing the ePRO‐naïve and the ePRO‐experienced feasibility survey groups. Due to one expected cell count less than five, a Fisher's exact test was performed in this case. Differences were considered as statistically significant at a *p* < .05. All statistical analyses were performed using SPSS 21.0.

## 3 RESULTS

### 3.1 Recruitment

**Figure 3:**
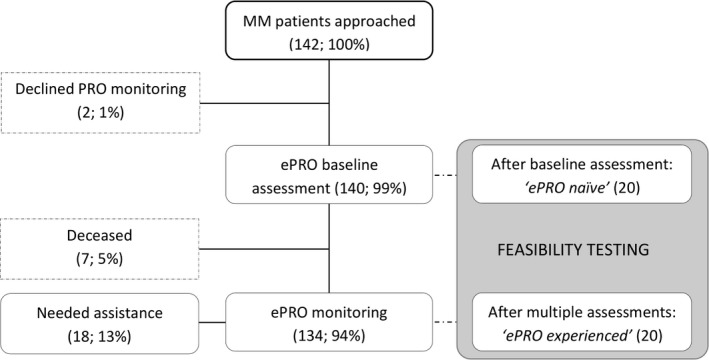
Flow chart of patient inclusion and subgroup analysis for feasibility testing

Between June 2016 and September 2017, 134/ 142 (94.4%) of the MM patients treated at the outpatient unit consented to participate in the AMR‐linked routine ePRO monitoring. 142 patients (100%) were approached, 140/ 142 (94.4%) patients provided consent for inclusion (two declined due to anticipated response burden) and six patients declined participation after first baseline assessment due to impaired general health condition (cf. Figure 3). Seven patients (5%) unfortunately deceased since the initiation of ePRO monitoring. At the time of analysis, about half of the patients included in ePRO monitoring completed the ePRO questionnaires two to five times (up to 22 times) resulting in an overall of 748 PRO assessment time points having been included in the registry. Thirteen per cent of the patients (18/134) included required assistance for questionnaire completion on the regular basis. Significantly more Internet non‐users required assistance for ePRO assessment than Internet users (*p* < .001; 11/52 vs. 1/68). This might be explained by the fact that Internet non‐users were significantly older than participants who indicated to be Internet users (mean 72.5, *SD* 9.2, 95% CI 70.1–75.1 vs. mean 59.1, *SD* 10.3, 95% CI 56.9–62.1; *p* < .001).

### 3.2 Sample characteristics

Patients participating in ePRO monitoring had a mean age of 65.4 years (*SD* 11.8) with about 60% being male. Mean age of patients deciding against participation in routine monitoring was higher (mean 74.5 years, *SD* 9.2, range 61–91) compared to patients participating (mean 64.9 years, *SD* 11.7, range 38–93). The majority of the patients providing consent (68.4%) indicated more than compulsory education. Sixty‐two per cent of the patients reported regular Internet usage for private and/ or professional purposes.

Mean time since diagnosis was 4.2 years, up to 26 years. The majority of the patients (68.4%) were undergoing active or maintenance therapy, being treated with chemo—or myeloma specific therapy such as proteasome inhibitors (e.g., bortezomib or carfilzomib), immune‐modulatory drugs (lenalidomomid, or pomalidomide), melphalan or bendamustin, or a combination with antibodies like Daratumumab. Twelve (8.5%) patients have received autologous stem cell transplantation since the initiation of the ePRO monitoring. Sociodemographic data, clinical characteristics and treatment details of patients participating in routine ePRO monitoring are summarised in Tables 1.

**Table 1:** Sample characteristics (*N* = 142)

Age	Mean 65.4 years (SD 11.8; 38–93 years)
Sex, *N* (%)	
Male	84 (59.2)
Female	58 (40.8)
Highest education, *N* (%)	
Less than compulsory	5 (3.5)
Compulsory	33 (23.2)
Apprenticeship/college	67 (47.2)
high school	12 (8.5)
University, academy	18 (12.7)
Missing	7 (4.9)
Non‐German mother tongue	14 (9.9)
Internet usage, *N* (%)	
No	52 (36.6)
Yes	88 (62.0)
Missing	2 (1.4)
Current treatment, *N* (%)	
Watch and wait (w & w)	22 (15.5)
Supportive care	23 (16.2)
Active treatment^a^	84 (59.2)
Maintenance therapy	13 (9.1)
Time since diagnosis	Mean 4.2 years (*SD* 4.8; 0–26.5 years)

a Incl. chemotherapy, autologous stem cell transplant etc.

The dataset was screened for missing responses to the scoring items. Across all time points (*N* = 748), the largest percentages of missing responses were observed for items assessing MM‐specific QOL issues: 4.1% (item 49: ‘Have you been worried about dying?’), 5.9% (item 36: ‘If you had pain did it increase with activity?’) and 6.7% (item 47: ‘Have you felt physically less attractive as a result of your disease or treatment?’). Because the item on hair loss is conditional, it was except from analysis of missing data. For all other items, missings did not amount to higher than 3.1%.

### 3.3 Evaluation of the feasibility and usability of routine ePRO monitoring

A subsample of 40 patients completed the feasibility questionnaire, 20 at baseline (‘ePRO‐naïve’) as well as after multiple (at least 5) assessment time points (‘ePRO‐experienced’) each (cf. Table 2). The two groups differed significantly with regard to the frequency of their outpatient visits (60% of ePRO‐naïve group every 3 to 6 months vs. 95% of ePRO‐experienced group weekly to monthly; *U* = 82.5, *p* < .001) and current treatment (active treatment in 55% vs. 85% patients; *U* = 304.5, *p* = .004). Twice as much participants in the ePRO‐experienced compared to the ePRO‐naïve group were male.

**Table 2:** Feasibility sample characteristics (*N* = 40)

	Total sample (*N* = 40)	ePRO‐naïve (*n* = 20)	ePRO‐experienced^a^ (*n* = 20)	*p*‐value
Age (years)	Mean 62.9 (*SD* 12.7)	Mean 64.7 (*SD* 11.0)	Mean 61.3 (*SD* 14.4)	.529
Sex, *n* (%)				
Male	19 (47.5%)	7 (35.0%)	12 (60.0%)	.113
Female	21 (52.2%)	13 (65.0%)	8 (40.0%)	
Internet usage, *n* (%)				
No	17 (42.5%)	8 (40.0%)	9 (45.0%)	.355
Yes	22 (55.0%)	11 (55.0%)	11 (55.0%)	
Missing	1 (2.5%)	1 (5.0%)	0 (0.0%)	
Highest education, *n* (%)				
Compulsory or less	8 (20.0%)	5 (25.0%)	3 (15.0%)	.095
Apprenticeship	24 (60.0%)	14 (70.0%)	10 (50.0%)	
High school/ university	8 (20.0%)	1 (5.0%)	7 (35.0%)	
Missing	0	0	0	
Frequency of outpatient visits, *n* (%)				
Every 6 months	6 (15.0%)	6 (30.0%)	0 (0.0%)	**<.001** *
every 3 months	7 (17.5%)	6 (30.0%)	1 (5.0%)	
Monthly	11 (27.5%)	3 (15.0%)	8 (40.0%)	
Every 2 weeks	4 (10.0%)	1 (5.0%)	3 (15.0%)	
Weekly or more	12 (30.0%)	4 (20.0%)	8 (40.0%)	
Treatment, *n* (%)				
w&w^b^	1 (2.5%)	1 (5.0%)	0 (0.0%)	**.035** *
w&w and support	6 (15.0%)	4 (20.0%)	2 (10.0%)	
Active (e.g., antibodies, chemotherapy)	28 (70.0%)	11 (55.0%)	17 (85.0%)	
Maintenance	5 (12.5%)	4 (20.0%)	1 (5.0%)	

a Equal to or more than five assessment time points per patient.

b w&w—watch and wait (surveillance).

* Bold values indicates *p* < .05

#### 3.3.1 Patients’ perspective on feasibility and usability of routine ePRO monitoring

About 87.5% (35/40) of the feasibility subsample reported not having problems with the usage of the tablet for ePRO assessment (85% in the ePRO‐naïve group; cf. Table 3). The software proved to be user‐friendly regarding the basic features necessary to complete a questionnaire (e.g., skip to next or return to previous question). When asked if questionnaire completion became easier after multiple assessments, patients’ responses varied with 65% (13/20) of the patients in the ePRO‐experienced group responding with ‘quite’ to ‘very much’. In this subsample, 42.5% reported not to use the Internet at all. No difference was found with regard to acceptability (*p* = .619) and requirement for assistance for ePRO (*p* = .082) between Internet users and non‐users.

**Table 3:** Patients’ perspective on usability of routine ePRO monitoring (*N* = 40)

	Total sample (*n* = 40)	ePRO‐naïve (*n* = 20)	ePRO‐experienced^a^ (*n* = 20)	*p*‐value
Problems with usage, *n* (%)				
Not at all	35 (87.5)	17 (85.0)	18 (90.0)	.607
A little	4 (10.0)	3 (15.0)	1 (5.0)	
Quite a bit/ very much	0 (0.0)	0 (0.0)	0 (0.0)	
Missing	1 (2.5)		1 (5.0)	
Need for assistance, *n* (%)				
Not at all	24 (60.0)	9 (45.0)	15 (75.0)	.123
A little	10 (25.0)	8 (40.0)	2 (10.0)	
Quite a bit	2 (5.0)	0 (0.0)	2 (10.0)	
Very much	2 (5.0)	2 (10.0)	0 (0.0)	
Missing	2 (5.0)	1 (5.0)	1 (5.0)	
Completion is easier after multiple assessments, *n* (%)				
Not at all	—	—	2 (10.0)	—
A little	—	—	4 (20.0)	
Quite a bit	—	—	6 (30.0)	
Very much	—	—	7 (35.0)	
Missing			1 (5.0)	
Know how to skip a question, *n* (%)				
No	3 (7.5)	1 (5.0)	2 (10.0)	.272
Yes	31 (72.5)	15 (75.0)	16 (80.0)	
Missing	6 (15.0)	4 (20.0)	2 (10.0)	
Know how to go back to previous question, *n* (%)				
No	3 (7.5)	0 (0.0)	3 (15.0)	.334
Yes	30 (75.0)	16 (80.0)	14 (70.0)	
Missing	7 (17.5)	4 (20.0)	3 (15.0)	
Know how to close questionnaire, *n* (%)				
No	2 (5.0)	0 (0.0)	2 (10.0)	.614
Yes	32 (80.0)	16 (80.0)	16 (80.0)	
Missing	6 (15.0)	4 (20.0)	2 (10.0)	

a Equal to or more than five assessment time points per patient.

The majority of the patients questioned (87.5%, 35/40) was quite to very satisfied with the ePRO monitoring procedure, while 80% (32/40) have not experienced any delay in clinical routine due to ePRO (cf. Table 4). Seventy‐seven point five per cent of the patients (31/40) perceived the questionnaire to be a good to very good method to inform the HCP about their subjective health state. To discuss their QOL data with their doctor was quite a bit to very important to 72.5% (29/40). No statistically significant difference was found between the ePRO‐naïve and the ePRO‐experienced group.

**Table 4:** Patient satisfaction with ePRO monitoring procedure and perceived value (*N* = 40)

	Total sample (*N* = 40)	ePRO‐naïve (*n* = 20)	ePRO‐experienced^a^ (*n* = 20)	*p*‐value
Satisfaction with monitoring, *n* (%)				
Not at all	0 (0.0)	0 (0.0)	0 (0.0)	.435
A little	1 (2.5)	0 (0.0)	1 (5.0)	
Quite a bit	13 (32.5)	8 (40.0)	5 (25.0)	
Very much	22 (55.0)	9 (50.0)	13 (65.0)	
Missing	4 (10.0)	3 (15.0)	1 (5.0)	
Perceived delay in clinical routine, *n* (%)				
Not at all	32 (80.0)	14 (70.0)	18 (90.0)	.356
A little	5 (12.5)	4 (20.0)	1 (5.0)	
Quite a bit	0 (0.0)	0 (0.0)	0 (0.0)	
Very much	1 (2.5)	0 (0.0)	1 (5.0)	
Missing	2 (5.0)	2 (10.0)	0 (0.0)	
Questionnaire as good method to inform doctor about health state, *n* (%)				
Not at all	0 (0.0)	0 (0.0)	0 (0.0)	.540
A little	9 (22.5)	5 (25.0)	4 (20.0)	
Quite a bit	6 (15.0)	1 (5.0)	5 (25.0)	
Very much	25 (62.5)	14 (70.0)	11 (55.0)	
Importance to talk about own QOL results with doctor, *n* (%)				
Not at all	6 (15.0)	3 (15.0)	3 (15.0)	.919
A little	5 (12.5)	2 (10.0)	3 (15.0)	
Quite a bit	9 (22.5)	5 (25.0)	4 (20.0)	
Very much	20 (50.0)	10 (50.0)	10 (50.0)	

a Equal to or more than five assessment time points per patient.

Regarding the schedule of data assessment, the majority of the 40 patients questioned on feasibility would prefer to complete the QOL questionnaire monthly, during treatment as well as in aftercare (cf. Table 5). The majority (16/20, 80%) of the ePRO‐experienced group was willing to spend up to 15 min, while 45% (9/20) of the ePRO‐naïve group would spend up to half an hour. The two groups differed significantly regarding preferred frequency of questionnaire completion during (*p* = .023) as well as after treatment (*p* = .037) and accepted length of completion time (*p* = .031). Patients at baseline assessment would prefer to complete the ePRO questionnaire significantly less often (75% monthly or less during treatment and 50% once to twice a year in aftercare) than patients after multiple assessments (90% weekly to monthly during treatment and 60% monthly in aftercare). When asked which location for questionnaire completed they would prefer if given the option, the majority of patients (24/40, 60%) indicated the clinic, while 32.5% did not report any preference at all.

**Table 5:** Patients’ preferences concerning ePRO completion logistics (*N* = 40)

	Total sample (*N* = 40)	ePRO‐naïve (*n* = 20)	ePRO‐experienced^a^ (*n* = 20)	*p*‐value
Preferred frequency of questionnaire completion, *n* (%)				
During treatment				
Never	1 (2.5)	0 (0.0)	1 (5.0)	**.037** *
Weekly	6 (15.0)	2 (10.0)	4 (20.0)	
Every 2 weeks	5 (12.5)	0 (0.0)	5 (25.0)	
Monthly	17 (42.5)	8 (40.0)	9 (45.0)	
Less often	8 (20.0)	7 (35.0)	1 (5.0)	
Missing	3 (7.5)	3 (15.0)	—	
After treatment, *n* (%)				
Never	2 (5.0)	0 (0.0)	2 (10.0)	**.023** *
Monthly	16 (40.0)	4 (20.0)	12 (60.0)	
Every 3 months	5 (12.5)	2 (10.0)	3 (15.0)	
Every 6 months	10 (25.0)	7 (35.0)	3 (15.0)	
Yearly	3 (7.5)	3 (15.0)	0 (0.0)	
Missing	4 (10.0)	4 (20.0)	—	
Preferred place of assessment, *n* (%)				
No preference	13 (32.5)	8 (40.0)	5 (25.0)	.472
At home	3 (7.5)	2 (10.0)	1 (5.0)	
Clinic	24 (60.0)	10 (50.0)	14 (70.0)	
Preferred way to receive questionnaire at home, *n* (%)				
Post	9 (22.5)	6 (30.0)	3 (15.0)	.322
Email	9 (22.5)	3 (15.0)	6 (30.0)	
No preference	7 (17.5)	2 (10.0)	5 (25.0)	
Missing	15 (37.5)	9 (45.0)	6 (30.0)	
Accepted length of time, *n* (%)				
0 min	1 (2.5)	1 (5.0)	0 (0.0)	**.031** *
1–15 min	21 (52.5)	5 (25.0)	16 (80.0)	
16–30 min	12 (30.0)	9 (45.0)	3 (15.0)	
46–60 min	1 (2.5)	1 (5.0)	0 (0.0)	
Missing	5 (12.5)	4 (20.0)	1 (5.0)	

a Equal to or more than five assessment time points per patient.

* Bold values indicates *p* < .05

#### 3.3.2 Healthcare professionals’ perspective on feasibility and usability of ePRO monitoring

Fifteen HCPs (four physicians, 11 nurses) participated in the survey on ePRO feasibility. Eighty per cent of these HCPs indicated being quite to very satisfied with ePRO monitoring, about 67% (10/15) did not experience any delay in clinical routine (cf. Table 6). Minor delays, indicated by 33.3% (5/15), were encountered in the clinical workflow due to completing the ePRO assessment in a separate room and proceeding to the clinical examination in another. Thirteen HCPs (86.7%) providing feedback valued the QOL data as a good method to receive information about patients’ health and considered it to be important to check the patients’ completed questionnaire. Sixty‐seven per cent of the HCPs (10/15) found it quite a bit to very important to talk to the patients about their QOL results, mostly if the patient's QOL was worse than expected (53.3%, 8/15) or has worsened since the last assessment time point (26.7%, 4/15).

**Table 6:** Healthcare providers’ perspective on feasibility (*N* = 15)

	*n* (%)
Questionnaire as good method to receive information about patients health	
Not at all	0 (0.0)
A little	2 (13.3)
Quite a bit	8 (53.3)
Very much	5 (33.3)
Importance to see patients’ QOL data	
Not at all	0 (0.0)
A little	5 (33.3)
Quite a bit	7 (46.7)
Very much	3 (20.0)
Satisfaction with routine monitoring	
Not at all/ a little	0 (0.0)
Quite satisfied	9 (60.0)
Very satisfied	3 (20.0)
Missing	3 (20.0)
Delay in clinical routine	
Not at all	10 (66.7)
A little	5 (33.3)
Quite a bit/ very much	0 (0.0)
Wish for feedback about patients QOL (yes/ no)	
Yes: 13 (86.7)	
Printout	3 (20.0)
Homepage	1 (6.7)
CIS^a^	8 (53.3)
Verbally	1 (6.7)
Importance of being confronted with patients’ perceptions of bad health state	
Not at all/ a little	0 (0.0)
Quite a bit	8 (53.3)
Very much	7 (46.7)
Importance to talk about results with patients	
Not at all	1 (6.7)
A little	4 (26.7)
Quite a bit	7 (46.7)
Very much	3 (20.0
Reasons to speak with patients about their QOL	
QOL < than expected	8 (53.3)
QOL worsened	4 (26.7)
Other reason	1 (6.7)
Missing	2 (13.3)
Preferred frequency of receiving QOL data	
During treatment	
Never	1 (6.7)
Weekly	2 (13.3)
Every 2 weeks	3 (20.0)
Monthly	6 (40.0)
Rarer	1 (6.7)
After treatment	
Never	3 (20.0)
Monthly	2 (13.3)
Every 3 months	6 (40.1)
Every 6 months	2 (13.3)
Yearly	2 (13.3)

a CIS clinical information system.

The majority (86.7%, 13/15) wished to receive feedback about their patients’ QOL, preferably as part of the clinic information system (reported by 53%, 8/15). If the patient was under active treatment, the majority of HCPs (60%, 9/15) considered feedback on QOL data to be necessary every two to four weeks (with the option of receiving feedback ‘per therapy cycle’). When asked for the preferred frequency for feedback about the patients’ QOL data after treatment completion, 40% (6/15) preferred data every 3 months, while 20% (3/15) indicated not to have any wish for feedback at all.

#### 3.3.3 Observational data on on‐site performance

Since initial operation (13th June 2016), logistic and technical issues observed on‐site refer to lack of privacy (no designated room) and to matters of surface disinfection when using a touch screen device in the healthcare setting. These were resolved in due course and supplemented with additional interventions fostering routine monitoring. Hence, measures to increase adherence with ePRO monitoring included adapting assessment frequency to patient preference and further customisation of the software to patient demands (i.e., the size of the icons especially for older patients with peripheral neuropathy).

## 4 DISCUSSION

This study aimed at depicting the implementation procedure and evaluating the feasibility of routine monitoring of ePROs for integration with data in the AMR at a single participating clinical centre. Adherence to routine monitoring was high, while the percentage of missing data proved to be low across all assessment time points. ePRO monitoring demonstrated feasibility in clinical practice, while undergoing further adaption and improvement based on stakeholder feedback.

A key challenge to successful ePRO monitoring is achieving high levels of sustained patient participation. Participation rates have been far beyond those reported in the literature (Ashley et al., 2013; Hutchings, Neuburger, Grosse Frie, Black, & van der Meulen, 2012; Smith et al., 2016; Thiel et al., 2015). Since patient participation is higher when patients are approached face‐to‐face (Ashley et al., 2013), the presence of the ePRO facilitator might have been a supporting factor. Even more so for 13% of the patient sample requiring assistance with ePRO assessment on a regular basis. Significantly more Internet non‐users seemed in need for support in handling the system than Internet users which might be due to the fact that the former were significantly older than participants who indicated to use the Internet regularly. This difference might resolve in the future with greater penetration of new technologies and generational shift.

Experiencing the treating HCP addressing issues from the ePRO data in real‐time strengthens patients’ motivation to participate further, resulting in a positive HCP–patient feedback loop. Likewise, it is also important to take into account the patient preference on assessment time points and completion frequency, which differed significantly between the ePRO‐naïve and the ePRO‐experienced group. The majority of the ePRO‐experienced group (80%) was willing to spend significantly less time (15 min) to answer the questionnaire, however, more often (90% weekly to monthly during treatment and 60% monthly in aftercare) then the ePRO‐naïve group. This might point towards a learning effect concerning questionnaire completion but also evaluating their own health status after multiple assessment time points as well as might correspond to the current treatment pathways of the respective patients. Supposedly, patients undergoing active treatment might experience more pronounced fluctuation in their health status than patients in maintenance therapy or under surveillance, have more frequent contact with their treating physicians and prefer receiving medical feedback regarding their QOL data.

Both patient groups, however, agreed that QOL questionnaires were a good method to report information to their physicians (75% vs. 80%) and that it was important to talk about their QOL data (75% vs. 70%). Feedback on QOL data is known to be an important aspect for patients’ adherence to QOL monitoring (Oerlemans, Arts, Horevoorts, & van de Poll‐Franse, 2017).

Among the HCPs, 87% considered receiving feedback on the patients’ QOL to be important and asked for feedback, preferably implemented in the CIS. HCPs would prefer being notified when the patients have indicated worsening of their QOL or when the patients reported worse QOL than the HPC would have expected. This corresponds with clinical reality and the conception of taking action when the patient's condition is in decline. ePRO monitoring system developers need to take this into account when designing software features for the QOL data feedback such as flag systems highlighting changes in a patients’ health state based on pre‐defined cut‐off values. Overall, these results mirror the literature available on HCPs’ attitudes towards the use of PRO data in clinical practice (Boyce, Browne, & Greenhalgh, 2014).

### 4.1 Lessons learned and action taken based on stakeholder evaluation

Empirical observations from clinical practice were used to both, expand theoretical knowledge about implementation and to take actual steps for adjustment. Based on patient feedback, the rhythm of ePRO monitoring was adjusted not only to treatment schedule but also to patient preference concerning completion frequency.

The importance of receiving patient data and also making it a subject of discussion with the patient was evident for 67% of the HCPs completing the feedback questionnaire. Given the importance of HCPs’ feedback for patient adherence and the benefits of routine monitoring for patient care (Basch et al., 2016), it is necessary to raise awareness for the availability of QOL data among healthcare providers. Hence, exchange was fostered at regular meetings and logistics adapted to support sustainability with routine procedures. On a more technical note, when assessing with touch screen devices, providers need to consider the locally existing guidelines on surface disinfection. Although regular disinfection with alcohol‐based wipes is recommended (Albrecht et al., 2013), we found this procedure to be detrimental for some devices. Alternative options would be using hydrogen peroxide wipes, UV‐light based approaches and asking patients to apply hand sanitisers before using the tablet.

### 4.2 Limitations

There are limitations to this study. The ePRO assessment has been limited to one outpatient unit providing data for the AMR. Considering the high patient turnover in an outpatient facility, the low dropout rates and low percentage of missing questionnaire data give way to the assumption that ePRO monitoring might be feasible in lower frequented wards (e.g., inpatient units) as well. However, there needs to be a person responsible for ePRO assessment, since approx. ten per cent will require assistance on a regular basis according to literature (Ala‐Aldeen et al., 2018). Composition of patient groups for feasibility survey was not balanced regarding their current treatment and consequently, regarding the frequency of their outpatient appointments. In the patient group solely completing baseline assessment, 55% have been undergoing active treatment, while 85% of the ePRO‐experienced patients in treatment were seen at the unit each week to month. This distribution is due to the study design based on consecutive recruitment: patients were enrolled during a period of 12 months; therefore, only the patients in active treatment with regular clinical appointments could become experienced in the ePRO monitoring.

### 4.3 Implications

The results presented herein have encouraged the extension of ePRO monitoring within the institution and on a multi‐institutional level. Driven by a local HCP initiative, further patient groups (according to cancer entity) and units have been included. Routine ePRO monitoring has been extended beyond the clinical setting by means of an online patient portal, where patients can access disease‐specific information, complete their QOL questionnaire and look at their QOL values that are linked to self‐management interventions. Next steps include optimising the logistics by integrating electronic reminders to enhance shared responsibility and buy‐in from the local HCPs to foster sustainable implementation into routine care. From a scientific standpoint, ePRO data are an important tool to validate differential treatment approaches from a patients perspective, especially in the chosen model of myeloma were multiple regimens and drugs without a clear oncological ranking or sequencing strategy compete for application.

### 4.4 CONCLUSION

Linking PROs with cancer registry data are essential for oncological treatment planning as well as for targeting quality assurance and research issues. ePRO monitoring can overcome the challenges of routinely collecting PROs for supplementing cancer registry data and has proven feasible. Our findings may provide useful insights for HCPs considering introducing ePRO monitoring to their units for informing clinical registries as well as individualised feedback to patients alike.

## CONFLICT OF INTEREST

Bernhard Holzner and Gerhard Rumpold hold IPRs on the software CHES. Wolfgang Willenbacher reports research funding from AMGEN, BMS, Celgene, Janssen, Novartis, Roche, Takeda and the participation in advisory boards/ committees and consultancies for AMGEN, BMS, Celgene, Gilead, Janssen, Novartis, Merck, Pfizer, Roche, Sandoz, Takeda; all outside the submitted work. None of the other authors has a conflict of interest to declare.

## ETHICAL APPROVAL

Ethical approval was obtained by the institutional review board (Innsbruck Ethics Committee, reference number AN 3252 266/4.2 386/5.17).
